# Protocol for genetic manipulation of cervical lymph node-innervating nociceptive neurons in mice via retrograde viral tracing

**DOI:** 10.1016/j.xpro.2026.104460

**Published:** 2026-03-18

**Authors:** Yibo Guo, Tong Ji, Yu Zhang

**Affiliations:** 1 Department of Oral and Maxillofacial Surgery, Zhongshan Hospital, Fudan University, Shanghai 200032, China; 2 Department of Stomatology, Zhongshan Hospital, Fudan University, Shanghai 200032, China; 3 Shanghai Stomatological Hospital & School of Stomatology, Fudan University, Shanghai 201102, China

**Keywords:** Cancer, Immunology, Model Organisms, Neuroscience

## Abstract

This protocol details the surgical procedure for genetically manipulating cervical lymph node (LN)-innervating nociceptive neurons in mice. We describe steps for exposing cervical LNs, microinjecting retrograde adeno-associated viruses (AAVs), and verifying viral transduction in the trigeminal ganglia. This approach enables specific labeling, chemogenetic inhibition, or gene knockout in LN-innervating neurons to study neuroimmune crosstalk.

For complete details on the use and execution of this protocol, please refer to Zhang et al.^1^

## Highlights

• Instructions for microsurgical exposure of mouse cervical lymph nodes
• Steps for precise retrograde viral microinjection into cervical nodes
• Guidance on verifying viral expression in trigeminal ganglia neurons

## Abstract (editor highlights)

Publisher’s note: Undertaking any experimental protocol requires adherence to local institutional guidelines for laboratory safety and ethics.

## Graphical abstract

**Figure:**
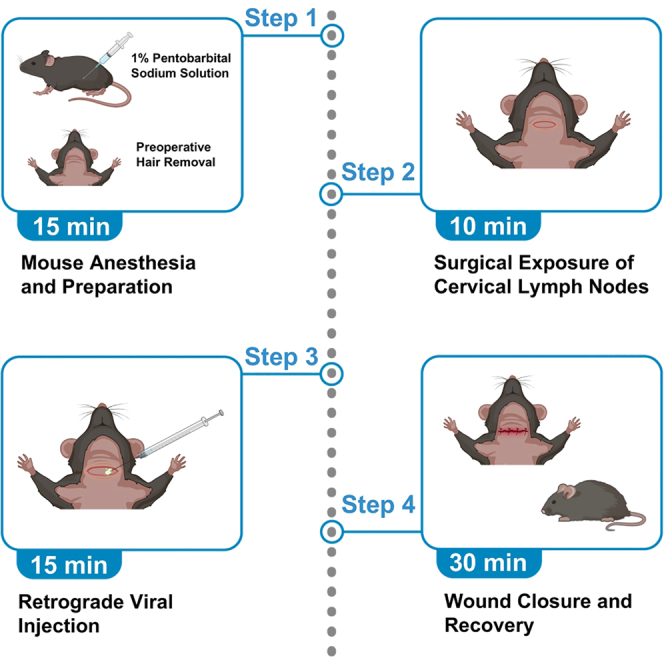

## Before you begin

The nervous system is a critical player in inter-organ immune regulation, and lymph nodes (LNs) are known to be innervated by sensory neurons with immunomodulatory potential.^2^ In the context of head and neck squamous cell carcinoma (HNSCC), the crosstalk between tumor-innervating nociceptive neurons and cervical tumor-draining lymph nodes (TDLNs) plays a pivotal role in remodeling the immune microenvironment. However, precisely manipulating these specific LN-innervating neurons without affecting the entire peripheral nervous system remains a technical challenge. Here, we present a protocol for the specific genetic manipulation of cervical LN-innervating nociceptive neurons in mice. Following the microinjecting retrograde adeno-associated viruses (AAV2/retro) directly into the cervical LNs, the virus undergoes retrograde transport to the neuronal cell bodies located in the trigeminal ganglia (TG). This approach allows for the specific labeling (e.g., using *hSyn*-EGFP), chemogenetic inhibition (e.g., using hM4D(Gi)-mCherry), or gene knockout (e.g., utilizing Cre-*loxP* systems) of these neurons to functionally dissect neuroimmune circuits. The protocol below describes the specific steps for using C57BL/6 mice. However, we have also used this protocol in genetically engineered strains, such as *Calca*^*flox/flox*^ and *Scn10a*^*CatCh-EYFP*^ mice.

### Innovation

This protocol introduces a high-precision methodology for genetically manipulating the specific subset of nociceptive neurons innervating cervical lymph nodes, addressing the limitations of systemic chemical denervation or global genetic models which lack spatial specificity. By utilizing AAV2/retro serotype vectors injected directly into the LNs, this protocol exploits retrograde transport to target distinct neuronal cell bodies within the trigeminal ganglia. This approach enables versatile functional dissections, including chemogenetic inhibition, optogenetic activation, and gene knockout. Furthermore, the workflow optimizes the microsurgical exposure technique to strictly preserve afferent lymphatic and blood vessels, ensuring optimal viral uptake. The integration of Fast Green visualization minimizes off-target leakage, establishing a reproducible platform to decode the tumor-TDLN neuroimmune axis.

While this protocol is optimized for cervical lymph nodes, the microsurgical exposure and injection techniques are readily adaptable to other superficial lymph nodes, such as the inguinal or axillary nodes. However, application to deep-lying lymph nodes (e.g., mesenteric lymph nodes) is associated with significantly higher surgical difficulty and mortality risks, necessitating advanced surgical skills and optimal field exposure.

### Institutional permissions

All animal care and experimental protocols involved in this study were strictly conducted in accordance with relevant ethical guidelines and were approved by the Institutional Animal Care and Use Committee of Zhongshan Hospital. All procedures were performed in compliance with the standards of the Regulations for the Administration of Experimental Animals, minimizing animal suffering and ensuring their welfare to the greatest extent.

**Figure 1:**
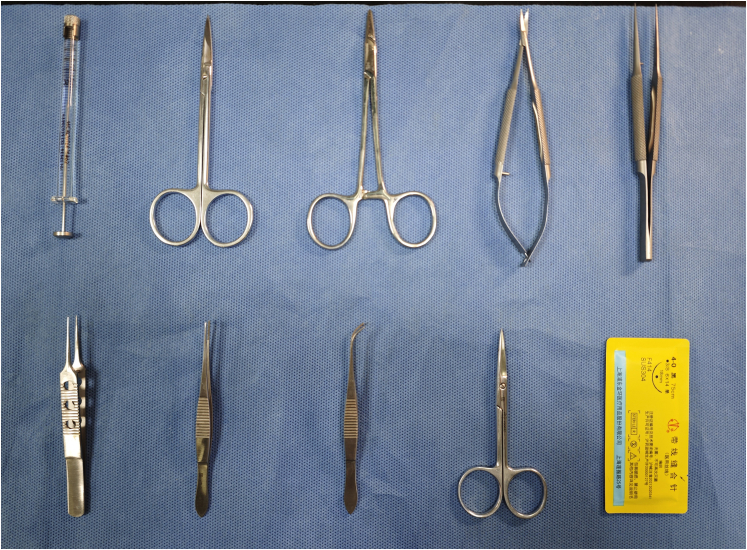
Partial surgical instruments and consumables display

### Preparation of viral vectors

**Timing: 10 min**

1. Retrieve the AAV aliquots from the −80°C freezer and place them on ice.
2. Allow the viral vectors to thaw completely.

**CRITICAL:** Use high-titer viruses to ensure sufficient retrograde transport efficiency. We recommend titers ≥ 1 × 10^13^ vg/mL. Lower titers (e.g., 5 × 10^12^ vg/mL) are acceptable but may result in fewer labeled neurons and reduced efficiency for functional manipulations.

***Note:*** Always keep the viral vectors on ice during the procedure to prevent titer degradation.

3. Prepare the 0.1% Fast Green solution.

**CRITICAL:** Pass the solution through a 0.22 μm filter to sterilize and remove any particulate matter that could clog the microsyringe.

4. Mix the thawed virus with the 0.1% Fast Green solution in a 3:1 ratio in a sterile microcentrifuge tube immediately prior to surgery.

**CRITICAL:** The Fast Green dye is essential for visualizing the spread of the injection within the lymph node and ensuring no leakage occurs.

***Note:*** Keep the viral mixture on ice until the moment of injection.

### Preparation of surgical instruments and consumables

**Timing: 10 min**

5. Cover the surgical table with a disposable surgical drape and systematically arrange the essential equipment for the procedure.

***Note:*** A dissecting microscope is recommended to ensure a clearer field of view for the cervical region, although the procedure can be performed without it.

6. Set up a heating pad on the operating table and pre-warm it to 37°C to maintain the mouse body temperature.
7. Arrange the anesthesia system (isoflurane vaporizer or injectable mix) and ensure it is functional.
8. Sterilize surgical instruments by high-pressure steam or a bead sterilizer prior to use.
9. Clean and disinfect the 2.5 μL Hamilton microsyringe carefully with 75% ethanol followed by sterile saline.

**CRITICAL:** Ensure the microsyringe plunger moves smoothly and is free of clogs to guarantee precise viral injection volume (troubleshooting-problem 4).

10. Prepare the necessary consumables and auxiliary supplies.

***Note:*** These include 75% ethanol for skin disinfection, depilatory cream for hair removal, sterile gauze, sterile saline (0.9% NaCl) for rinsing the injection site, ophthalmic ointment to prevent corneal drying, sterile cotton swabs, and 4-0 surgical sutures for wound closure (Figure 1).

***Note:*** Aseptic operation is of vital importance. The operating area should be disinfected before surgery, and disposable consumables must be used only once.

## Key resources table

**Table:** 

REAGENT or RESOURCE	SOURCE	IDENTIFIER
**Bacterial and virus strains**		

AAV2/retro-*hSyn*-EGFP	This paper	N/A

**Chemicals, peptides, and recombinant proteins**		

Pentobarbital sodium, 10 g	Hangzhou Dawen Bio	N/A
Fast Green FCF Stain Solution, 0.1%	Solarbio	G1660
Isoflurane, 100 mL	RWD Life Science	R510-22

**Experimental models: Organisms/strains**		

C57BL/6: Wild-type, 4–6 weeks old, male and female	Shanghai Model Organisms Center	N/A

**Other**		

Sodium chloride solution, 1,000 mL: 9 g	Sichuan Kelun	N/A
Lidocaine hydrochloride injection, 10 mL: 0.5 g	Harbin Sanma	N/A
Erythromycin eye ointment	Guangzhou Baiyunshan	N/A
Depilatory cream	Veet	N/A
Medical gauze swab, 10 cm × 10 cm, 8p	Zhende Medical	N/A
Medical tape	3M	1527C-1
4-0 Surgical suture	Jinhuan Medical	F401
0.22 μm filter	Millipore	SLGPR33RB
Microsyringe, 2.5 μL	Hamilton	7632–01

## Step-by-step method details

The total hands-on time for the surgical procedure is approximately 60 min per mouse for an experienced researcher.

### Anesthesia and preparation of the mouse

**Timing: 10 min**

This step ensures the mouse is properly anesthetized and the surgical site is prepared to minimize infection risk.

1. Select a healthy C57BL/6 mouse (4–6 weeks of age) and weigh the mouse to determine the appropriate anesthetic dosage.
2. Induce anesthesia using one of the following methods:
  a. Isoflurane inhalation:
    i. Place the mouse in an induction chamber with 3%–4% isoflurane (flow rate 0.8–1 L/min).
    ii. Once the mouse is recumbent, maintain anesthesia via a nose cone at 1.5%–2% isoflurane.
  b. Sodium pentobarbital injection:
    i. Firmly restrain the mouse and disinfect the lower abdomen injection site with 75% ethanol.
    ii. Insert the needle into the lower abdominal quadrant, aspirate the syringe to verify that there is no blood, intestinal fluid, or urine.
    iii. Gradually administer the 1% pentobarbital sodium solution.

***Note:*** Calculate the injection volume of sodium pentobarbital based on the mouse's body weight (typically 30–50 mg/kg).

***Note:*** Isoflurane inhalation is recommended due to its superior controllability and stability during delicate microsurgery.

**Figure 2:**
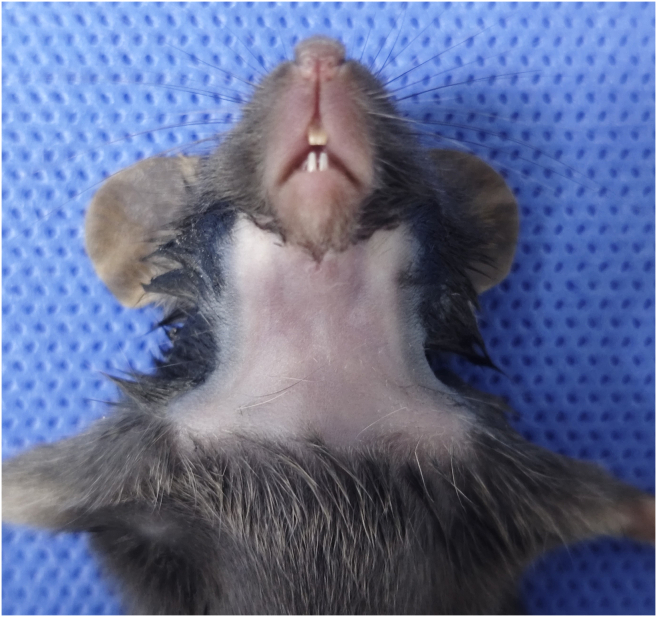
Pre-operative preparation of the surgical site

3. Return the mouse to its cage (if injected) or keep on the nose cone (if inhaled) and wait for the anesthetic to take full effect.
4. Verify the depth of anesthesia by pinching the hind paw; the absence of a withdrawal reflex indicates sufficient anesthesia.
5. Prepare the surgical site:
  a. Apply ophthalmic ointment to the eyes to prevent corneal drying.
  b. Place the mouse in a supine position on the heating pad to maintain body temperature.
  c. Secure the limbs with tape if necessary.
  d. Shave the fur from the submandibular region extending down to the root of the neck.
  e. Apply a depilatory cream for 1–2 min to remove remaining fine hair, then wipe clean with wet gauze.
  f. Disinfect the surgical site with povidone-iodine and 75% ethanol (Figure 2).

### Surgical exposure of cervical lymph nodes

**Timing: 10 min**

This step exposes the cervical lymph nodes for viral injection. Crucially, it preserves the afferent lymphatic vessels and blood supply to ensure effective retrograde transport to the trigeminal ganglia.

6. Gently flatten the skin of the mouse’s neck to identify the mid-cervical surgical region.
7. Administer a small amount of lidocaine subcutaneously at the planned incision site to provide local anesthesia.
8. Under the dissecting microscope, make a 1–1.5 cm midline incision in the neck skin using surgical scissors (troubleshooting-problem 1).
9. Use micro-forceps to gently separate the subcutaneous connective tissue and salivary glands to expose the underlying cervical lymph nodes.

***Note:*** The cervical LNs are typically located superficial to the sternocleidomastoid muscles, often in pairs on each side.

**Figure 3:**
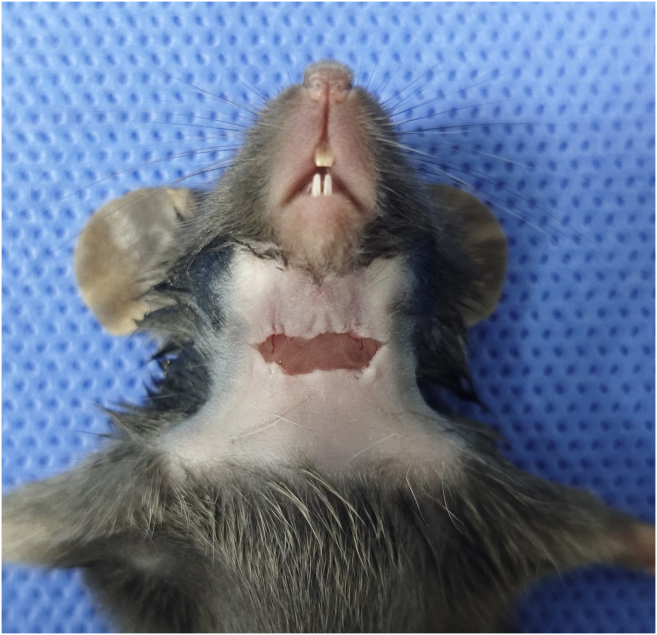
Midline cervical incision

10. Carefully dissect the thin fascia surrounding the target lymph node using blunt dissection with micro-forceps (Figure 3).

**CRITICAL:** Perform this step with extreme caution. Do not damage the afferent lymphatic vessels or surrounding blood vessels. Hemorrhage or lymphatic disruption will impair viral uptake and transport.

***Note:*** If significant bleeding occurs, apply gentle pressure with a sterile cotton swab until hemostasis is achieved.

### Retrograde viral injection

**Timing: 15 min**

This step details the precise microinjection of the viral vector into the exposed cervical lymph nodes. Achieving accurate volume delivery and visual confirmation via Fast Green dye are critical to ensure targeted transduction of the innervating nociceptive neurons while strictly minimizing off-target leakage.

11. Carefully withdraw the prepared viral mixture (Virus + Fast Green) into the 2.5 μL Hamilton microsyringe.

***Note:*** Ensure absolutely no air bubbles are trapped within the syringe barrel or needle, as injecting air can disrupt the lymph node architecture and block viral uptake.

12. Stabilize the lymph node gently using micro-forceps.

**CRITICAL:** Grasp the surrounding adipose tissue or connective tissue to stabilize the node. Do not pinch the lymph node capsule directly, as this may cause mechanical damage or rupture.

**Figure 4:**
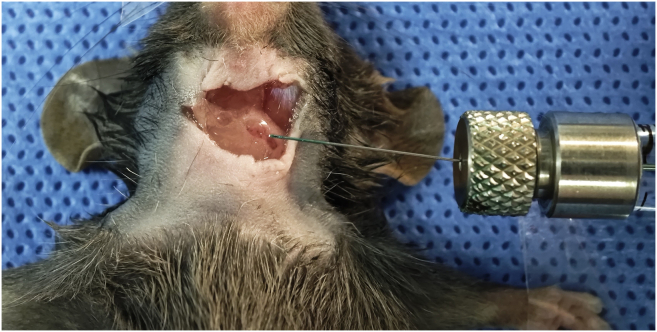
Microinjection of viral vector into the cervical lymph node

13. Insert the needle tip of the microsyringe into the center of the lymph node at a shallow angle (approximately 15°–20°) to maximize the needle track length within the node (Figure 4) (troubleshooting- problem 2).

***Note:*** Ensure the needle bevel is facing upward. This orientation facilitates smoother tissue penetration and minimizes the risk of accidentally puncturing the posterior capsule of the lymph node.

14. Slowly inject the viral mixture over a period of 0.5–1 min to prevent rapid expansion and rupture of the lymph node.

***Note:*** For labeling/tracing: Inject 2 μL of the mixture (1.5 μL AAV + 0.5 μL Fast Green) into the unilateral cervical LN of 4–6-week-old mice. Reduce the volume for younger mice or smaller nodes to prevent rupture.

***Note:*** Successful injection is confirmed by the lymph node turning visibly green due to the Fast Green dye. If the dye leaks extensively into the surrounding tissue immediately, the injection may have failed (troubleshooting-problem 3).

15. Leave the needle in place for an additional 30 s after the injection is complete.

***Note:*** This pause allows the viral fluid to diffuse within the tissue and reduces backflow along the needle track upon withdrawal.

**Figure 5:**
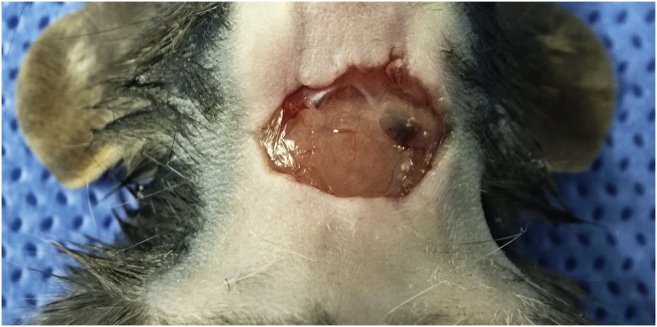
Visual confirmation of successful intranodal injection

16. Slowly withdraw the needle and immediately rinse the injection site and surrounding tissue with 1 mL of sterile saline (Figure 5).

**CRITICAL:** This wash step is vital to remove any leaked virus and prevent non-specific infection of surrounding muscle fibers or cutaneous nerves.

### Wound closure and recovery

**Timing: 25 min**

This step focuses on restoring the anatomical integrity of the cervical skin and ensuring the safe recovery of the animal.

**Figure 6:**
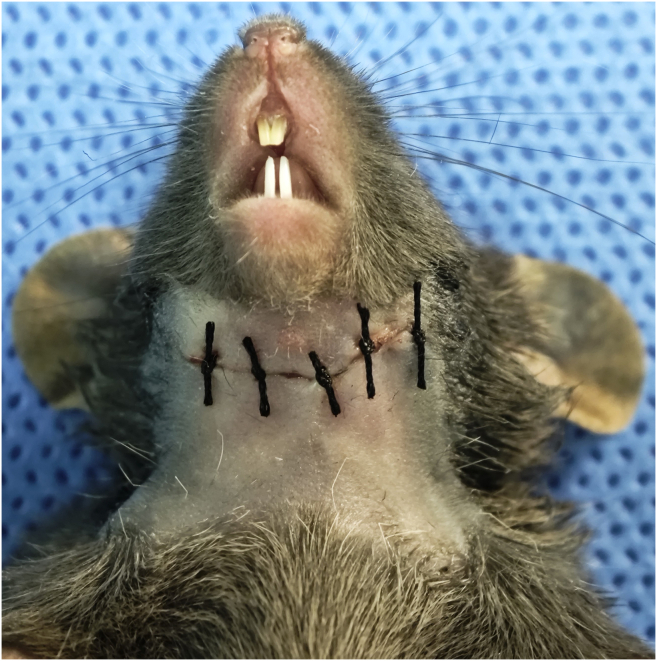
Wound closure of the cervical incision

17. Verify there is no active bleeding from the surgical site or surrounding tissues.
18. Suture the skin incision with 4-0 non-absorbable sutures (using interrupted stitches) (Figure 6).

**CRITICAL:** Insert the needle close to the skin edge (approximately 1–2 mm from the incision margin). Do not suture too deeply or tightly, as excessive tension or bulky suturing can restrict neck extension, impeding the mouse's ability to raise its head and feed properly.

19. Apply topical antibiotic ointment and lidocaine cream directly to the incision site to prevent infection and provide local analgesia.
20. Remove the mouse from the anesthesia system and place it in a clean, pre-warmed recovery cage.

***Note:*** Do not return the animal to its home cage until it has fully regained consciousness and mobility to prevent hypothermia or injury from cage mates.

21. Monitor the mouse until it is fully awake and ambulatory (typically 20–30 min) before returning it to the home cage (troubleshooting-problem 5).

***Note:*** Monitor the mice daily for 3 days post-surgery for signs of pain, distress, or infection.

## Expected outcomes

Upon successful execution of this protocol, retrograde viral expression should be observed in the ipsilateral (for unilateral injection) or bilateral (for bilateral injection) trigeminal ganglia (TG) approximately 6 weeks post-injection. The primary site of viral transduction will be the neuronal cell bodies, confirming the effective retrograde transport from the cervical lymph nodes.

**Figure 7:**
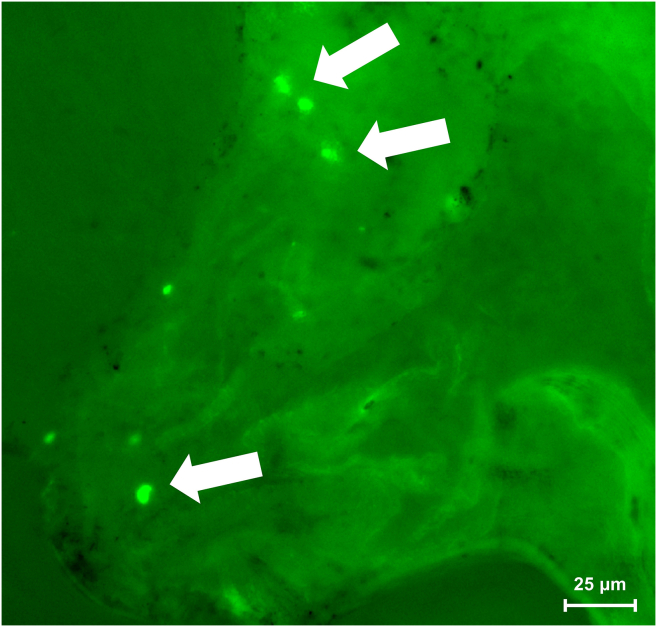
Retrograde viral expression in the trigeminal ganglia

Taking the retrograde labeling virus (AAV2/retro-*hSyn*-EGFP) as an example, researchers can expect to see bright EGFP fluorescence in a distinct subset of TG neurons (Figure 7). If other viral vectors are used, such as those for chemogenetic inhibition or gene knockout, corresponding verification methods should be employed. Immunofluorescence analysis reveals that approximately 30% of the EGFP-positive neurons co-localize with Calcitonin Gene-Related Peptide (CGRP),^1^ confirming their identity as peptidergic nociceptive neurons. The labeling should be specific to the neurons innervating the injected lymph node, with minimal to no labeling in contralateral TGs in unilateral models or non-relevant sensory ganglia.

***Note:*** These outcomes are based on experiments in C57BL/6 mice. If applying this protocol to other species (e.g., rats), we recommend conducting pilot experiments to verify neuronal labeling efficiency.

## Limitations

The primary limitation of this protocol lies in the stringent requirement for microsurgical precision. The structural integrity of the delicate afferent lymphatic vessels and the surrounding vascular network must be strictly preserved during lymph node exposure; any accidental disruption or hemorrhage can sever the retrograde transport pathway, resulting in experimental failure. Additionally, this method necessitates a prolonged incubation period to ensure sufficient retrograde transport and robust transgene expression in the trigeminal ganglia. This extended timeline limits the applicability of the protocol for experimental designs requiring rapid temporal resolution. Although AAV2/retro expression has been reported as early as 3 weeks,^3^ we recommend a 6-week incubation period to ensure robust transgene levels; shorter durations may yield insufficient expression for functional studies.

Furthermore, cervical lymph nodes receive dual innervation from sensory and autonomic neurons. While this protocol focuses on the nociceptive pathway, the sympathetic nervous system also plays a critical role in modulating the immune microenvironment. To investigate these autonomic contributions, this protocol can be adapted by utilizing viral vectors driven by the tyrosine hydroxylase (TH) promoter, allowing for the independent dissection of sympathetic inputs. Finally, pathological states such as infection or tumor metastasis significantly alter the lymph node microenvironment. These changes may impact viral diffusion and uptake by nerve endings; therefore, researchers should anticipate potential variability in labeling efficiency when applying this protocol to disease models.

## Troubleshooting

### Problem 1

Accidental damage to the subcutaneous lymph nodes or surrounding vasculature during the initial skin incision (related to step 8).

### Potential solution

Sufficient exposure without damaging the lymph node structural integrity and surrounding vessels is key to success. Beginners often fail to control the incision depth or struggle to balance exposure with tissue preservation, leading to either difficult injections or vascular damage. To avoid accidental injury, use micro-forceps to gently lift the skin of the mid-cervical region away from the underlying tissue before making the incision. This creates a safety gap between the skin and the delicate subcutaneous structures. Additionally, making a small initial nick and then extending the incision with blunt scissors can further minimize the risk. We recommend a training period of at least 5–10 mice to master these microsurgical skills; operators with prior clinical surgical experience may acquire proficiency more rapidly.

### Problem 2

Mechanical rupture of the lymph node capsule or needle penetration through the node (related to step 13).

### Potential solution

This issue frequently arises when the needle insertion angle is too steep or excessive force is applied during entry. A steep trajectory may cause the needle to pierce through the posterior capsule of the small cervical lymph node, while excessive force can disrupt the structural integrity of the organ. To prevent this, approach the lymph node at a shallow angle with the needle bevel facing upward. Ensure the needle tip remains within the central parenchymal region. If the lymph node is visually observed to be pierced through or severely ruptured, the animal should be excluded from the study as the viral uptake will be compromised.

### Problem 3

Viral leakage into perinodal tissues, evidenced by the rapid spread of Fast Green dye (related to step 14).

### Potential solution

Leakage of the viral vector typically occurs due to high intranodal pressure caused by rapid injection or backflow along the needle track upon withdrawal. This results in non-specific labeling of surrounding muscle fibers or cutaneous nerves, confounding the interpretation of specific innervation. To minimize this risk, inject the viral mixture extremely slowly. After the injection is complete, leave the needle in place for at least 30 s to allow for pressure equilibration and fluid diffusion. Upon needle withdrawal, immediately and thoroughly rinse the injection site with sterile saline to wash away any residual dye or virus before closing the incision.

To validate specificity, we recommend examining non-draining ganglia (e.g., dorsal root ganglia); in our experiments, these tissues showed no viral expression when the injection was strictly confined to the node, serving as a negative control for off-target effects.

### Problem 4

Obstruction of the microsyringe needle or plunger, preventing fluid aspiration or injection (related to before you begin-step 9).

### Potential solution

This issue is typically caused by residual precipitates accumulating in the syringe barrel due to inadequate cleaning between procedures, or by undissolved particles in self-prepared Fast Green solutions. To prevent this, thoroughly clean the Hamilton microsyringe before and after each use by repeatedly aspirating and ejecting 75% ethanol followed by sterile saline to remove all residues. If preparing the Fast Green solution from powder, ensure it is fully dissolved and pass it through a 0.22 μm filter before mixing it with the viral vector to eliminate any particulate matter that could block the fine-gauge needle.

### Problem 5

Unexpected mortality of mice during the perioperative period (related to step 21).

### Potential solution

Postoperative mortality is commonly attributed to anesthesia overdose, uncontrolled hemorrhage, or impaired feeding caused by restrictive cervical suturing. To mitigate these risks, strictly adhere to body-weight-based anesthetic dosing and ensure complete hemostasis of major cervical vessels before closure. Avoid placing sutures too deeply or tightly, as this can restrict neck extension and prevent proper feeding. Maintain thermal support until full recovery of consciousness and monitor body weight daily; if significant weight loss occurs, provide soft food or hydrogel to support recovery.

### Problem 6

No viral expression observed in the trigeminal ganglia despite surgical success.

### Potential solution

First, verify the viral titer and storage conditions. AAV vectors are sensitive to repeated freeze-thaw cycles; ensure aliquots are stored at −80°C and kept on ice during the procedure. Second, the injection may have been restricted to the subcapsular space or surrounding adipose tissue rather than the nodal parenchyma. Ensure the needle tip penetrates the center of the lymph node. Third, in certain transgenic strains, immune clearance of the virus or labeled neurons may occur; we recommend validating the viral efficacy in wild-type C57BL/6 mice first.

## Resource availability

### Lead contact

Further information and requests for resources and reagents should be directed to and will be fulfilled by the lead contact, Yu Zhang (zhangyu_zsyy@fudan.edu.cn).

### Technical contact

Further information and requests for technical issues should be directed to and will be fulfilled by the technical contact, Yibo Guo (guoyibo@fudan.edu.cn).

### Materials availability

This study did not generate new unique reagents.

### Data and code availability

This study did not generate any unique datasets or code.

## Acknowledgments

This work was supported by the following grants: 10.13039/100016698National Natural Science Foundation of China (82403363, 82372862, and 82173147), China National Postdoctoral Program for Innovative Talent (BX20240091), 10.13039/501100002858China Postdoctoral Science Foundation (2024M750549), 10.13039/100007219Natural Science Foundation of Shanghai Municipality (24ZR1411700), Outstanding Resident Clinical Postdoctoral Program of 10.13039/501100010108Zhongshan Hospital Affiliated to 10.13039/501100003347Fudan University (2025ZYYS-035 and 2025ZYYS-067), and 10.13039/100017633Shanghai Anticancer Association EYAS PROJECT (SACA-CY25B03).

## Author contributions

Y.G. and Y.Z. helped with protocol optimization and wrote the manuscript. T.J. participated in the design of the protocol and provided valuable suggestions.

## Declaration of interests

The authors declare no competing interests.
